# A Proposal of Utilizing Six Types of Involvement Model to Guide Kindergarten to 12th Grade School Parental Communication and Support During a Pandemic

**DOI:** 10.18103/mra.v12i4.5178

**Published:** 2024-04-26

**Authors:** Dan Li, Yueqi Li, Ziyi Zheng, Xin Zhou, Danielle Castro, Sten H. Vermund, Marie A. Brault

**Affiliations:** 1 Yale School of Public Health, Yale University, New Haven, CT, United States; 2 Harvard Graduate School of Education, Cambridge, MA, United States; 3 School of Social Policy and Practice, University of Pennsylvania, PA, United States; 4 NYU Grossman School of Medicine, New York, NY, United States

## Abstract

**Background:**

Effective communication between schools and parents Is crucial for fostering understanding, trust, and collaboration to enhance educational outcomes and student well-being, especially during crises such as the COVID-19 pandemic. Moreover, the current level of communication between schools and families is frequently insufficient, exacerbating the difficulties in parental engagement, comprehension, and certain policy implementation. This deficiency becomes even more pronounced during crises due to the added stressors. This study aims to highlight the challenges of parental engagement and communication during the pandemic and propose a viable solution for school districts and schools to enhance trust, understanding, and collaboration in schools to prepare for future crises.

**Method:**

The study employs a mixed-methods approach, Including a scoping review of literature and policies on school communication during the pandemic, a survey study conducted among the Connecticut Independent Schools, and the Integration of results from both sources. The scoping review provides key themes and frameworks, while the survey collects quantitative and qualitative data to identify challenges and concerns. The proposed solution utilizes Epstein’s Six Types of Involvement Framework for school districts and schools to guide effective communication and collaboration between schools and parents.

**Results:**

The scoping review and survey findings reveal several key Issues, Including hesitant parental perception of disease control strategies, the burden on parents in supporting online learning, the lack of resources and guidance for online learning, and the absence of central communication guidelines. The proposed solution, Epstein’s Six Types of Involvement Framework, addresses these challenges by emphasizing parenting, communication, volunteering, learning at home, decision-making, and community collaboration.

**Conclusion:**

The study highlights the importance of effective communication between schools and parents during crises and proposes Epstein’s Six Types of Involvement Framework as a comprehensive solution. By implementing this framework, schools can foster understanding, trust, and collaboration, leading to better educational outcomes for students. The findings have implications for school administrators, policymakers, and educators seeking to improve communication during crises and can facilitate more effective communication and parental engagement beyond health crises. Further research Is needed to evaluate the effectiveness and impact of implementing the framework in real-world crises. Moreover, healthcare professionals like pediatricians, psychologists, and school nurses are crucial in disease control in schools. The study proposes using Epstein’s framework to Involve them directly, enhancing collaboration and trust, and empowering them to lead efforts in safeguarding students and staff health.

## Introduction

Effective communication between schools and parents is a vital component for fostering understanding, trust, and collaborative efforts toward enhancing educational and developmental outcomes, especially during a crisis. Despite its importance, existing school-home communication is far from optimal. A survey conducted among Kindergarten to 12th grade public school teachers in the United States revealed that 50% of Kindergarten to 12th grade public school teachers rated parental involvement in their children’s education as inadequate, with 48% reporting inadequate parental understanding of the curriculum (Ozmen et al., 2016). The challenges posed by the COVID-19 pandemic have further compounded this gap in communication, highlighting an urgent need to develop comprehensive communication guidance and model. In this study, the pivotal roles of health professionals in facilitating effective communication channels between schools and parents during crises like the COVID-19 pandemic cannot be overstated. Their expertise in health communication, empathy, and understanding of diverse community needs are instrumental in bridging gaps and fostering resilience in educational environments.

The purpose of this paper is to identify key challenges of parental engagement and communication during the COVID-19 pandemic, to provide a viable solution to enhance trust and understanding and foster better support and collaboration in schools during the current and future crises. In section IV, the paper commences with a literature and policy analysis exploring the difficulties in parental support and school communication during the COVID-19 crisis in the United States. We supplement this document review with survey and interview data from 6th-12th grade (or middle and high) schools in Connecticut. In section V, we propose to develop a solution that entails the development of a communication framework to guide school districts and administrators to communicate effectively and expeditiously with different stakeholders, addressing the discrepancy in perceptions and needs. Our findings will be most relevant to school administrators and politicians seeking to communicate effectively during a crisis like the COVID-19 pandemic. It may also facilitate more effective communication and parental engagement outside of health crises.

## Methods

In this study, we used multiple sources of information to outline the school-parental communication challenges and proposed a potential solution for better communication during a crisis like the pandemic. First, we conducted a scoping review and extracted key themes from studies related to Kindergarten to 12th grade school communication challenges (Section 111 A). Second, we used survey tools to investigate concerns related to the pandemic and communication for members of the Connecticut Independent Schools during the pandemic (Section NIB). Third, we integrated the results from the scoping review and findings from the survey to outline Kindergarten to 12th grade school-parental communication challenges and proposed Epstein’s Six Types of Involvement framework as an interventional construct to guide and aid communication (Section IIIC). Through these three interconnected components, our study aims to provide insights into the school-family communication processes brought about by the COVID-19 crisis and offer practical recommendations for supporting the well-being of school members during a crisis. Below are the detailed methods employed in each of the three parts of the study:

### A. SCOPING REVIEW

We conducted a scoping review on Kindergarten to 12th grade schools in the United States, whose protocol details have been published previously (Li et al., 2021). Articles related to school communication and pandemic perceptions were identified and key results and themes were summarized. First, we focused on and outlined school-family challenges in Section IV [Issues], In addition, we reviewed existing school-based communication frameworks and selected Epstein’s Six Types of Involvements as our interventional construct in Section V [Solution].

### B. SURVEY STUDY

#### B.1 Participant and Context

This survey was conducted from October 2020 to April 2021 in private schools affiliated with the Connecticut Association of Independent Schools (CAIS). The following were the inclusion criteria for parents and staff respondents: (1) English-speaking; (2) having consent for study participation. Investigators had private access to data via encrypted drives.

#### B.2 Survey Instruments

Surveys used the Coronavirus Safety Behaviors Scale, the Preventive COVID-19 Behavior Scale, and the Johns Hopkins University COVID-19 Community Response Survey to measure their perceived COVID risks and attitudes towards preventative behaviors and policies. The survey questions and responses are presented in Table 2–4. We also included open-ended questions for respondents to submit their concerns and comments.

#### B.3 Survey Administrations and Enrollment

The study was approved by Yale School Public Health’s Institutional Review Board (#2000028873). These survey tools were emailed to eligible participants using Qualtrics^®^ software to ensure anonymity and data privacy. We conducted three rounds of surveys for parents and staff. The initial survey round was administered between October and November 2020, while data for the second round was collected in January 2021, and the final round was conducted in April 2021. There are a total of 1784 staff and 13974 parents who were invited to participate in the study. 609, 41, and 66 parents responded to survey rounds one, two, and three, respectively. 309, 22, and 15 staff responded to survey rounds one, two, and three, respectively. The demographics information of the parent and staff survey participants are presented in Appendix II.

#### B.4 Quantitative Analysis

We used descriptive statistics to describe the basic features of the data collected. We analyzed the distribution, central tendency, dispersion, and pattern of each variable. For descriptive purposes, we expressed categorical variables as proportions and continuous variables as means, medians, and standard deviations (SD). We made comparisons between answers in surveys that were administered at different time points, and between parent and staff surveys, using χ^2^ and fisher’s exact test, as appropriate, with statistical significance defined as p<0.05 (2-tailed. We similarly summarized respondents’ characteristics using descriptive statistics.

#### B.5 Qualitative Analysis

The responses to open-ended questions were reviewed and coded using Microsoft Word^®^ to determine patterns and constructs through thematic analysis. This analysis is guided by the following steps according to Guest et. al., (2012): (1) familiarization, and organization of responses; (2) identification of themes; (3) review and analysis of themes to determine structures or constructs and; (4) construction of the theoretical model vis-à-vis new data. It sought to unpack constructs (Vaismoradi et. al, 2013) of the Reach, Effectiveness, Adoption, Implementation, and Maintenance framework (RE-AIM) that is contextualized at private schools’ issues and approaches towards communication and concerns amid COVID-19.

### C. INTEGRATION OF THE RESULTS

In our integrated approach, we combined the results from our survey study, key findings from the scoping review, and our interventional assessment, to provide a comprehensive analysis that can inform school policies. The mixed-methods fashion allowed us to provide a more complete picture of school-parent communication issues. We identified four key issues from our analysis of literature reviews, surveys, and open-ended questions: (a) hesitant perception among parents regarding disease control strategies; (b) the burden of disease control strategies for parents; (c) lack of guidance and resources for schools to establish communication and; (d) lack of timely communication. With the findings in mind, we propose a solution that is anchored to Epstein’s six types of involvement framework to strengthen partnerships between communities, families, and schools towards better collaboration vis-à-vis communication.

## Results

## ISSUE I- PARENTS HOLD A MORE HESITANT PERCEPTION OF DISEASE CONTROL STRATEGIES THAN STAFF

We found a discrepancy in knowledge and attitudes toward the pandemic and disease-control strategies between school staff and parents. At the beginning of the pandemic, although school closure was widely adopted to prevent the spread of the pandemic, as demonstrated in 130 out of 193 countries (Daniela et al., 2021), many parents posited a negative attitude towards closures and certain disease control policies. Our survey showed that, compared to school staff, parents are more hesitant to trust the effectiveness of various disease prevention measures. Notably, 14% of parents believed that closing schools has no impact on disease prevention, while only 4% of staff members held this opinion. Moreover, while only 10% of parents agree that school closures are extremely effective in preventing the spread of disease, 13% of staff members share this perspective (Table 2–3). A greater proportion of school staff members believe that closing gyms, shops, and restaurants, wearing masks, and avoiding crowds and public spaces are effective means to prevent the spread of disease, in contrast to parents (Table 4). More staff than parents believes that mandatory mask wearing can help prevent the transmission of COVID-19 (Table 5). The reasons behind such disagreements are multifarious, ranging from beliefs that the illness is mild and school closures are unwarranted to doubts regarding the effectiveness of school closures in curbing infection rates (Brooks et al., 2020).

Different perceptions and attitudes towards disease control strategies can lead to disagreements, which could potentially exacerbate parental disengagement, particularly in the absence of effective communication between schools and families, preventing the formation of alliance and cooperation during the crisis, and causing more family-school conflicts. Therefore, the key to getting everyone on the same page and avoiding conflict is to understand different stakeholders’ knowledge and perceptions and to address any discrepancy using an effective communication strategy.

## ISSUE II- CERTAIN DISEASE CONTROL STRATEGIES PUT A HEAVY BURDEN ON PARENTS.

Since the beginning of the COVID-19 outbreak, many nations have kept their schools closed to control the virus. This has infected more than 1.6 billion students globally (United Nations International Children’s Emergency Fund (UNICEF) data hub, 2022). In response, many schools transferred in-person teaching to online learning to ensure students were still getting their formal education by studying at home. However, this sudden transition has not been without its challenges, and parents have had to take on additional responsibilities to supervise their children’s education (Doyle 2020).

Parental educational obligations are essential to help with online learning since young children will probably need help from caregivers to access and complete online learning materials (Lau et al., 2021). A majority of parents express worry and low self-efficacy in sufficiently supporting children through remote learning (Doyle 2020; Daniela et al., 2021). This is partly because children’s assignments are often too difficult for children to finish independently and require more parental support than they could offer (Jones & Forster, 2021). Furthermore, parents may feel that they lack the expertise to help with certain subjects (Daniela et al., 2021).

Parents are not only struggling to cope with the pandemic’s health risks such as food shortage, loss of income, economic downturn, and health threats, but they also have to dedicate time and energy to support their children’s education. This added pressure has taken a toll on parents’ mental health, with many reporting a negative impact (Gadermann et al., 2021). Research completed recently during the initial COVID-19 shutdown in the United States, indicates that parents may face higher levels of stress if they have a more challenging time promoting their children’s academic success (SpinelIi et al., 2020). Therefore, it is imperative to develop an effective school-home communication strategy that addresses the challenges faced by parents and other stakeholders. The provision of resources is also critical to ensure that parents are equipped to support their children’s learning.

## ISSUE III: LACK OF RESOURCES AND GUIDANCE REGARDING ONLINE LEARNING EXACERBATES THE CHALLENGING SITUATION

One of the fundamental requirements for successful online learning is technological competence. Parents have to familiarize themselves with novel digital tools to help their children access class materials, which is especially challenging for those parents who don’t usually use digital devices. Parents have not received adequate technical support from schools (Abuhammad, 2020). For example, they did not get guidance on how to use computers, what to do if the connectivity is poor, or how to access the learning portal. There has been a dearth of technical personnel to assist during class time or after class. Furthermore, some families lack the financial means to purchase electronic devices such as computers, cameras, and microphones. Dependable technology could significantly enhance the online learning experience by reducing the need for parents to repeatedly address technical difficulties.

These challenges are especially pronounced for families who lack access to computers and stable internet connections, such as those from low socioeconomic backgrounds and/or those living in rural areas. Nearly 7% of European families lack internet access and 5% of families don’t have places for children to do homework. In areas that encounter COVID-19 spikes like New York City, 10% of children experience either homeless situations or housing instability (Van Lanker and Parolin, 2020). Families with single parents, sick parents, or illiterate parents need schools’ extra support in children’s learning (Daniela et al., 2021). School administrators and staff should keep track of families’ living conditions and adjust the learning materials to ensure accessibility for all students.

## ISSUE IV: LACK OF CENTRAL COMMUNICATION GUIDELINES

SARS-CoV-2 variants evolved rapidly in their transmission, morbidity, and mortality rates. Policymakers and educators were faced with the challenge of updating policies and guidelines, and communicating these updates promptly (Sullivan et al. 2021). Federal and state government guidelines focus primarily on safety protocols. It is difficult for school boards to remain up-to-date with developing and revised CDC guidelines (Sullivan et al. 2021). There is often a flood of inconsistent and ever-changing information that school members (staff, parents, and students) receive. After a protocol has been developed, its implementation requires the participation and cooperation of all school members.

It is disappointing that, despite its critical and fundamental role, no guide instructs school districts on how to communicate and engage parents, staff, and community members during these uncertain times. Communication advice is vague and of no immediate benefit to the already overburdened schools. As a result, schools will have to develop their structure and content for communicating in addition to the tasks associated with the pandemic. In the absence of central guidance, self-directed communication is inefficient, incomplete, and error-prone. Consequently, inconsistent and incoherent messages can exacerbate mistrust, myths, and confusion, undermining the overall effectiveness of the school’s efforts.

The success of any protocol or guideline implementation requires the participation and cooperation of all school members, especially during a crisis. Therefore, school members’ knowledge, understanding, trust, attitudes, and concerns are crucial to the success of these efforts. To this end, a structured and comprehensive model for communication and engagement is essential during a time of crisis. The current mission and challenges faced by schools further emphasize the need for such a model to be developed and implemented urgently for schools and school districts to utilize.

## Discussion

In order to solve these challenges, we proposed schools and school districts use Epstein’s Six Types of Involvement Framework to guide communication. Epstein’s Six Types of Involvement Framework is one of the most influential models of communication and engagement in school, family, and community settings (Zagellari & Mipo, 2020). Developed in the early 1990s by Joyce Epstein and her collaborators, the Framework of Six Types of Involvement-sometimes known as the “School-Family-Community Partnership Model” has undergone revisions over the years, though the fundamental elements have remained unchanged (Williams et al., 2022): parenting, communicating, volunteering, home-based learning, decision-making, and collaborating with the community.

Below, we have outlined how each component of the Six Types of Involvement Framework can be applied to improve communication and engagement during times of crisis (Table 1):

1. Parenting: School professionals should have a basic understanding of the diversity of students and families. They need to keep track of families’ backgrounds, situations, and concerns and need to adequately assist caregivers during the transition period to at-home learning. Teachers could collect information such as parents’ confidence in supporting students’ learning, needs for technology support, parenting skills training, and any financial /familiaI hardship they are experiencing. This information can guide school professionals to provide corresponding accommodations and empowerment to families, including but not limited to multiple ways of displaying class content and student engagement (live sessions, video recording, posts on websites, phone messages) and diverse parental workshops.
2. Communicating: Families and school teachers should find an effective way of communication to ensure prompt delivery of school policy updates and students’ progress reports. For example, a school can utilize digital report cards regularly, multiple ways of notice (e.g. phone calls, newsletters, messages), or set up online conferences with parents to update school policies about the pandemic and convey teachers’ feedback on students’ learning. Clear communication is critical for all stakeholders to build accurate expectations for the unfamiliar remote learning and reach a consensus on school policies, which could help foster companionship among the school and families.
3. Volunteering: In terms of having families on the same page with schools, families are suggested to be involved in school projects or plans. As part of the project or plan, they would understand the background information well and know details on how to act. Throughout the process, a bridge is built between families and schools. Involved families could deliver the information and school goals to less involved families. Schools could have a new perspective on families’ needs and improve families’ engagement in future projects. Additionally, medical professionals can contribute by volunteering their time and expertise to support school initiatives, such as health and wellness programs or informational sessions for families.
4. Learning at home: Teachers could provide brochures or digital handouts for caregivers that specifically describe how to support students’ learning. This guidance may include expectations for parents’ involvement in remote learning (e.g. encouraging, guiding, monitoring), homework policies, and skills or habits parents can help students foster. For example, during the pandemic crisis, teachers could help families build stress management skills or conduct social-emotional learning at home, to buffer the negative effect of the pandemic on students’ mental health. Medical health professionals can offer expertise in areas such as stress reduction techniques, mental health support, and guidance on maintaining overall well-being during remote learning periods.
5. Volunteering: In terms of having families on the same page with schools, families are suggested to be involved in school projects or plans. As part of the project or plan, they would understand the background information well and know details on how to act. Throughout the process, a bridge is built between families and schools. Involved families could deliver the information and school goals to uninvolved families. Schools could have a new perspective on families’ needs and improve the families’ engagement for further projects launch or development. Medical health professionals could also play a role in volunteering their time or expertise to support school initiatives, such as health and wellness programs or informational sessions for families on topics related to physical and mental health.
6. Collaborating with the community: The school’s connection to the local community goes beyond resource procurement; it involves actively engaging with community voices. Schools play a crucial role in influencing the local economy, tourism, and industry. Therefore, fostering open forums to listen and collaborate with the community is essential for building sustainable relationships. Within this collaborative framework, medical professionals in the community emerge as invaluable allies. They offer insights into prevalent health needs, provide essential resources and referrals, and actively contribute to addressing health-related challenges through collaborative efforts.

Overall, Epstein’s Six Types of Involvement Framework provides a comprehensive approach to building and strengthening partnerships between schools, families, and communities. By addressing different types of involvement, the framework emphasizes the importance of communication, collaboration, and understanding, including the valuable contributions of medical professionals in supporting students and families during times of crisis.

### STRENGTHS AND LIMITATIONS

There are multiple strengths to our study and approach. Firstly, the study utilized the mixed method approach and provided a comprehensive array of data related to communication perceptions and behaviors of parents and faculty about various facets of COVID-19 risk and concerns. Second, our study proposed a practical model to address the challenges and concerns.

There are several limitations. The current participation rate in this particular study is notably low compared to the desired level of engagement. This could limit the generalizability of our findings. Additionally, the Multi-tiered System of Supports (MTSS) framework that we propose has not been specifically tested during the COVID-19 pandemic, although it has been implemented successfully in other school settings. The existing model has not undergone comprehensive testing or evaluation to assess its effectiveness and impact on the various stakeholders involved. To ensure successful implementation and sustainability, rigorous testing methods should be employed to evaluate the outcomes of this framework.

### IMPLICATIONS FOR SCHOOLS

From the scoping review and the survey, we found that parents hold hesitant perceptions of disease control strategies, with disagreements arising from beliefs about the severity of the illness and doubts regarding the effectiveness of school closures. Parents are more hesitant to trust disease prevention measures compared to school faculty, which can lead to conflicts and disengagement. School closures place a burden on parents who must support online learning and face challenges in providing sufficient assistance. Lack of resources and guidance for online learning exacerbates the situation, especially for families without access to technology and stable internet connections. Additionally, the absence of central communication guidelines hinders effective communication between schools and parents, resulting in inconsistent and confusing messages. Urgent attention is needed to address these issues through effective communication strategies, the provision of resources, and the development of comprehensive models for communication and engagement during crises.

The proposed utilization of six types of involvement models to guide Kindergarten to 12th grade school parental communication and support during a pandemic has significant implications for the physical, mental, and public health of school communities. The COVID-19 pandemic disrupted traditional schooling, and schools had to adopt new approaches to ensure that students continue to receive quality education. Since parental involvement is critical to the success of these new approaches, this proposal provides a framework for schools to engage parents in a meaningful way. By using the six types of involvement framework, schools can ensure that parents are informed, empowered, and involved in their children’s education during the pandemic.

The use of Epstein’s six types of involvement framework recognizes the role and contribution of school, community, and parents for better attainment of learning outcomes. As such, community and parent volunteer participation in school activities strengthens school programs and partnerships to improve school-community health and learning. In this view, parental involvement is essential to create policies, especially in monitoring student progress during the pandemic. Although the current health crisis significantly reduced the students’ social interaction, the resilience of parental and guided intervention offers better behavioral and emotional engagement (Asanjarani et al., 2023). With Epstein’s involvement framework, parents may gain awareness and self-confidence because of the increased comfort of conducting school activities at home. Volunteering efforts provide an arena of interaction with families in the community that enables sharing of practices toward the quality attainment of student learning. Moreover, communication and parenting are strategies to engage parents in utilizing information and following the pedagogical guidelines situated in the COVID-19 pandemic. Parents are highly influential in motivating learners to succeed academically (Wang & Sheikh-Khalil, 2014).

The significance of teacher-parent communication in raising children’s academic achievement and highlighting effective cooperation and communication between parents and teachers to guide children’s abilities will enhance learning outcomes (Spear et al., 2023). Teachers and facilitators understood the importance of parents’ involvement in helping students with course selection, building connections, tracking progress, encouraging learning activities, scheduling learning time, and offering study tips and topic support. Parents and teachers both had challenges, nevertheless, which were also noted (Borup et al., 2019). In particular with “hard-to-reach” families, schools made great efforts to retain communication and assistance, which enhanced family-school connections (Jones & Forster, 2021).

Parental involvement is a form of facilitation of remote instruction (learning at home) because they are involved in encouraging their children to engage in homework while parents gain an understanding of the school program. Future studies are needed to test the implementation of the model in real-world crises.

As researchers, we also stress the importance of medical professionals in understanding and addressing the challenges faced by parents during crises like the COVID-19 pandemic. By using engagement models, they can effectively communicate with and empower parents. Advocating for improved communication and resource allocation can enhance the well-being of school communities during crises.

## Conclusion

In conclusion, effective communication between schools and parents plays a crucial role in fostering understanding, trust, and collaborative efforts to enhance educational outcomes, particularly during times of crisis such as the COVID-19 pandemic. However, current school-home communication is often inadequate, as evidenced by surveys and data indicating challenges in discrepancies in perceptions and expectations. The COVID-19 pandemic has further highlighted the need for comprehensive communication guidance and models to bridge the communication gap and promote effective support and collaboration in schools. By utilizing Epstein’s Six Types of Involvement Framework, schools can establish a comprehensive approach that considers the diverse needs of families and promotes collaboration. By focusing on parenting, communication, volunteering, learning at home, decision-making, and community collaboration, schools can foster understanding, trust, and cooperation during times of rapid change and massive needs. The findings and proposed solution are particularly relevant for school administrators, policymakers, and educators seeking to improve communication during crises, and have the potential to facilitate more effective communication and parental engagement beyond health crises. By addressing these challenges and implementing the suggested framework, schools can strengthen their partnerships with parents, leading to better welfare for students.

Moreover, the main audience of this study is the medical professionals, including pediatricians, infectious disease doctors, and school nurses, who play a crucial role in communication strategies within educational settings. Their expertise in child health, disease transmission, and student health needs is essential for developing effective communication protocols that prioritize health and safety in schools. Recognizing and emphasizing their pivotal role in discussions about communication during crises is fundamental for promoting academic success and safeguarding the well-being of students and communities.

## Supplementary Material

1

## Figures and Tables

**Table 1.** Utilize Epstein’s Six Types of Involvement for Communication during a Crisis

	Parenting	Communicating	Volunteering	Learning at home	Decision Making	Collaborating
**Assess**	- Short survey to access school members’ knowledge - Short survey to access school members’ attitudes - Short survey to access school members’ behaviors - Short survey to access school members’ concerns - Open-ended assessment in school members’ concerns	- Collect and analyze school stakeholders’ knowledge - Collect and analyze school stakeholders’ attitudes - Collect and analyze school stakeholders’ behaviors - Collect and analyze school stakeholders’ concerns - Ensure two-way communications	- Put a list of needed task and activities - Collect a list of activities and tasks	- Assess children’s needs at home (resources, technologies, mental health, etc.)	- Host town hall meetings to brainstorm with different stakeholders	- Send school representatives to local community meetings - Engage in conversations with local community representatives
**Communication Resources and Channels**	- Townhall - Feedback collect platform - Focused Group	- Email templates and routines - Platform for immediately messaging - Website - Videos explaining different concepts - Articles/Papers explaining protocols - Guidelines and FAQ documents	- Sign-up sheets - Volunteer instruction - Activity protocols - Put parents in different cohort, assign different responsibilities to different cohort	- Establish manual highlight different home activities	- Invite different school stakeholders to attend meetings - Set up a voting system for different policies	- Engage in conversations with community leaders - Set up voting system - Establish email routines and material
**Engagement**	- Parental representative to participate in weekly planning Meetings		- Invite parents to different service projects		- Ask representatives of different stakeholders	- Invite community partners to provide resources - Invite community partners to engage in service projects

**Table 2.** Kindergarten to Grade 12 Parent and Staff Perceived COVID-19 Risks

	**Parent 1**		**Parent 2**		**Parent 3**		**P Values**
**Your child will be infected (n1 = 607; n2 = 39; n3 = 121)**							**1.892e-07**
**Answer**	**%**	**Count**	**%**	**Count**	**%**	**Count**	
No chance	0.49%	3	2.56%	1	3.31%	4	
Very small chance	32.78%	199	51.28%	20	48.76%	59	
Medium chance	55.35%	336	41.03%	16	34.71%	42	
High chance	6.1%	37	5.13%	2	2.48%	3	
Very high chance	2.97%	18	0.00%	0	0.83%	1	
Absolutely sure	0.33%	2	0.00%	0	0.00%	0	
This has already happened	1.98%	12	0.00%	0	9.92%	12	
	**Staff 1**		**Staff 2**		**Staff 3**		**P Values**
**You will be infected on your school’s campus (n1 = 380; n2 = 23; n3 = 32)**							**0.0093**
**Answer**	**%**	**Count**	**%**	**Count**	**%**	**Count**	
No chance	1.32%	5	4.35%	1	3.13%	1	
Very small chance	47.63%	181	60.87%	14	75%	24	
Medium chance	42.11%	160	30.43%	7	12.5%	4	
High chance	6.05%	23	0.00%	0	6.25%	2	
Very high chance	2.37%	9	4.35%	1	0.00%	0	
Absolutely sure	0.00%	0	0.00%	0	0.00%	0	
This has already happened	0.53%	2	0.00%	0	3.13%	1	
	**Parent 1**		**Parent 2**		**Parent 3**		**P Values**
**Someone in your child’s direct environment (family, friends, colleagues) will be infected (n1 = 606; n2 = 39; n3 = 121)**							**0.0089**
**Answer**	**%**	**Count**	**%**	**Count**	**%**	**Count**	
No chance	0.33%	2	0.00%	0	0.00%	0	
Very small chance	17.66%	107	23.08%	9	31.4%	38	
Medium chance	42.57%	258	43.59%	17	33.88%	41	
High chance	20,96%	127	12.82%	5	15.7%	19	
Very high chance	6.6%	40	2.56%	1	2.48%	3	
Absolutely sure	2.64%	16	0.00%	0	0.83%	1	
This has already happened	9.24%	56	17.95%	7	15.7%	19	
	**Staff 1**		**Staff 2**		**Staff 3**		**P Values**
**Someone in your direct environment (family, friends, colleagues) will be infected (n1 = 381; n2 = 23; n3 = 32)**							**0.054**
**Answer**	**%**	**Count**	**%**	**Count**	**%**	**Count**	
No chance	0.26%	1	0	0	0	0	
Very small chance	19.95%	76	21.74%	5	50%	16	
Medium chance	50.39%	192	47.83%	11	34.38%	11	
High chance	14.44%	55	17.39%	4	12.5	4	
Very high chance	6.82%	26	0.00%	0	0.00%	0	
Absolutely sure	1.84%	7	0.00%	0	0.00%	0	
This has already happened	6.3%	24	13.04	3	3.13%	1	
	**Parent 1**		**Parent 2**		**Parent 3**		**P Values**
**Your child will have to go to the hospital if they get the infection (n1 = 606; n2 = 39; n3 = 121)**							**<0.005**
**Answer**	**%**	**Count**	**%**	**Count**	**%**	**Count**	
No chance	9.41%	57	17.95%	7	11.57%	14	
Very small chance	68.48%	415	56.41%	22	70.25%	85	
Medium chance	16.5%	100	20.51%	8	11.57%	14	
High chance	2.97%	18	2.56%	1	2.48%	3	
Very high chance	0.5%	3	0	0	3.31%	4	
Absolutely sure	2.15%	13	2.56%	1	0.83%	1	
This has already happened	0.00%	0	0.00%	0	0.00%	0	
	**Staff 1**		**Staff 2**		**Staff 3**		**P Values**
**You will have to go to the hospital if you get the infection (n1 = 380; n2 = 23; n3 = 32)**							**<0.005**
**Answer**	**%**	**Count**	**%**	**Count**	**%**	**Count**	
No chance	2.11%	8	0.00%	0	9.38%	3	
Very small chance	48.68%	185	52.17%	12	56.25%	18	
Medium chance	34.21%	130	34.78%	8	21.88%	7	
High chance	8.16%	31	4.35%	1	6.25%	2	
Very high chance	5.79%	22	4.35%	1	0	0	
Absolutely sure	1.05%	4	4.35%	1	6.25%	2	
This has already happened	0.00%	0	0.00%	0	0.00%	0	
	**Parent 1**		**Parent 2**		**Parent 3**		**P Values**
**Your child will have to go into quarantine independent of them being infected or not (n1 = 603; n2 = 39; n3 = 120)**							**2.75E-06**
**Answer**	**%**	**Count**	**%**	**Count**	**%**	**Count**	
No chance	1%	6	2.56%	1	4.17%	5	
Very small chance	16.58%	100	15.38%	6	28.33%	34	
Medium chance	35.49%	214	46.15%	18	31.67%	38	
High chance	24.05%	145	17.95%	7	13.33%	16	
Very high chance	11.11%	67	0.00%	0	0.83%	1	
Absolutely sure	2.82%	17	7.69%	3	3.33%	4	
This has already happened	8.96%	54	10.26%	4	18.33%	22	
	**Staff 1**		**Staff 2**		**Staff 3**		**P Values**
**You will have to go into quarantine independent of you being infected or not (n1 = 379; n2 = 23; n3 = 32)**							**0.012**
**Answer**	**%**	**Count**	**%**	**Count**	**%**	**Count**	
No chance	0.53%	2	0.00%	0	0.00%	0	
Very small chance	15.83%	60	30.43%	7	43.75%	14	
Medium chance	40.63%	154	34.78%	8	25%	8	
High chance	21.11%	80	26.09%	6	18.75%	6	
Very high chance	12.14%	46	4.35%	1	0.00%	0	
Absolutely sure	3.69%	14	0.00%	0	9.38%	3	
This has already happened	6.07%	23	4.35%	1	3.13%	1	
	**Parent 1**		**Parent 2**		**Parent 3**		**P Values**
**Your child will get infected and they will infect someone else (n1 = 607; n2 = 39; n3 = 121)**							**5.34E-09**
**Answer**	**%**	**Count**	**%**	**Count**	**%**	**Count**	
No chance	1%	6	2.56%	1	4.17%	5	
Very small chance	16.58%	100	15.38%	6	28.33%	34	
Medium chance	35.49%	214	46.15%	18	31.67%	38	
High chance	24.05%	145	17.95%	7	13.33%	16	
Very high chance	11.11%	67	0.00%	0	0.83%	1	
Absolutely sure	2.82%	17	7.69%	3	3.33%	4	
This has already happened	8.96%	54	10.26%	4	18.33%	22	
	**Staff 1**		**Staff 2**		**Staff 3**		**P Values**
**You will get infected and you will infect someone else (n1 = 380; n2 = 23; n3 = 32)**							**0.085**
**Answer**	**%**	**Count**	**%**	**Count**	**%**	**Count**	
No chance	1.32%	5	4.35%	1	3.13%	1	
Very small chance	41.58%	158	43.48%	10	62.5%	20	
Medium chance	45.53%	173	43.48%	10	18.75%	6	
High chance	07.11%	27	4.35%	1	12.5%	4	
Very high chance	3.16%	12	4.35%	1	0.00%	0	
Absolutely sure	0.53%	2	0.00%	0	0.00%	0	
This has already happened	0.79%	3	0.00%	0	3.13%	1	
	**Parent 1**		**Parent 2**		**Parent 3**		**P Values**
**Someone in your child’s direct environment (family, friends, teachers) will die (n1 = 607; n2 = 39; n3 = 121)**							**0.10**
**Answer**	**%**	**Count**	**%**	**Count**	**%**	**Count**	
No chance	5.93%	36	2.56%	1	8.26%	10	
Very small chance	66.56%	404	69.23%	27	62.81%	76	
Medium chance	20.43%	124	17.95%	7	16.53%	20	
High chance	3.29%	20	2.56%	1	2.48%	3	
Very high chance	1.65%	10	0	0	2.48%	3	
Absolutely sure	0.16%	1	0	0	1.65%	2	
This has already happened	1.98%	12	7.69%	3	5.79%	7	
	**Staff 1**		**Staff 2**		**Staff 3**		**P Values**
**You will get infected and you will infect someone else (n1 = 380; n2 = 23; n3 = 32)**							**0.085**
**Answer**	**%**	**Count**	**%**	**Count**	**%**	**Count**	
No chance	1.32%	5	4.35%	1	3.13%	1	
Very small chance	41.58%	158	43.48%	10	62.5%	20	
Medium chance	45.53%	173	43.48%	10	18.75%	6	
High chance	7.11%	27	4.35%	1	12.5%	4	
Very high chance	3.16%	12	4.35%	1	0.00%	0	
Absolutely sure	0.53%	2	0.00%	0	0.00%	0	
This has already happened	0.79%	3	0.00%	0	3.13%	1	

	**Parent 1**		**Staff 1**		**P Values**
**Your child will be infected (n1 = 607; n2 = 380)**					**7.52E-05**
**Answer**	**%**	**Count**	**%**	**Count**	
No chance	0.49%	3	1.32%	5	
Very small chance	32.78%	199	47.63%	181	
Medium chance	55.35%	336	42.11%	160	
High chance	6.1%	37	60.5%	23	
Very high chance	2.97%	18	2.37%	9	
Absolutely sure	0.33%	2	0.00%	0	
This has already happened	1.98%	12	0.53%	2	
	**Parent 2**		**Staff 2**		**P Values**
**Your child will be infected (n1 = 39; n2 = 23)**					**0.49**
**Answer**	**%**	**Count**	**%**	**Count**	
No chance	2.56%	1	4.35%	1	
Very small chance	51.28%	20	60.87%	14	
Medium chance	41.03%	16	30.43%	7	
High chance	5.13%	2	0.00%	0	
Very high chance	0.00%	0	4.35%	1	
Absolutely sure	0.00%	0	0.00%	0	
This has already happened	0.00%	0	0.00%	0	
	**Parent 3**		**Staff 3**		**P Values**
**You will be infected on your school’s campus (n1 = 121; n2 = 32)**					**0.035**
**Answer**	**%**	**Count**	**%**	**Count**	
No chance	3.31%	4	3.13%	1	
Very small chance	48.76%	59	75%	24	
Medium chance	34.71%	42	12.5%	4	
High chance	2.48%	3	6.25%	2	
Very high chance	0.83%	1	0.00%	0	
Absolutely sure	0.00%	0	0.00%	0	
This has already happened	9.92%	12	3.13%	1	
	**Parent 1**		**Staff 1**		**P Values**
**Someone in your child’s direct environment (family, friends, colleagues) will be infected (n1 = 606; n2 = 381)**					**0.054**
**Answer**	**%**	**Count**	**%**	**Count**	
No chance	0.33%	2	0.26%	1	
Very small chance	17.66%	107	19.95%	76	
Medium chance	42.57%	258	50.39%	192	
High chance	20.96%	127	14.44%	55	
Very high chance	6.6%	40	6.82%	26	
Absolutely sure	2.64%	16	1.84%	7	
This has already happened	9.24%	56	6.3%	24	
	**Parent 2**		**Staff 2**		**P Values**
**Someone in your child’s direct environment (family, friends, colleagues) will be infected (n1 = 39; n2 = 23)**					**0.96**
**Answer**	**%**	**Count**	**%**	**Count**	
No chance	0.00%	0	0.00%	0	
Very small chance	23.08%	9	21.74%	5	
Medium chance	43.59%	17	47.83%	11	
High chance	12.82%	5	17.39%	4	
Very high chance	2.56%	1	0.00%	0	
Absolutely sure	0.00%	0	0.00%	0	
This has already happened	17.95%	7	13.04%	3	
	**Parent 3**		**Staff 3**		**P Values**
**Someone in your direct environment (family, friends, colleagues) will be infected (n1 = 121; n2 = 32)**					**0.25**
**Answer**	**%**	**Count**	**%**	**Count**	
No chance	0.00%	0	0.00%	0	
Very small chance	31.4%	38	50%	16	
Medium chance	33.88%	41	34.38%	11	
High chance	15.7%	19	12.5%	4	
Very high chance	2.48%	3	0.00%	0	
Absolutely sure	0.83%	1	0.00%	0	
This has already happened	15.7%	19	3.13%	1	
	**Parent 1**		**Staff 1**		**P Values**
**Your child will have to go to the hospital if they get the infection (n1 = 606; n2 = 380)**					**<0.005**
**Answer**	**%**	**Count**	**%**	**Count**	
No chance	9.41%	57	2.11%	8	
Very small chance	68.48%	415	48.68%	185	
Medium chance	16.5%	100	34.21%	130	
High chance	2.97%	18	8.16%	31	
Very high chance	0.5%	3	5.79%	22	
Absolutely sure	2.15%	13	1.05%	4	
This has already happened	0.00%	0	0.00%	0	
	**Parent 2**		**Staff 2**		**P Values**
**Your child will have to go to the hospital if they get the infection (n1 = 39; n2 = 23)**					**0.097**
**Answer**	**%**	**Count**	**%**	**Count**	
No chance	17.95%	7	0.00%	0	
Very small chance	56.41%	22	52.17%	12	
Medium chance	20.51%	8	34.78%	8	
High chance	2.56%	1	4.35%	1	
Very high chance	0	0	4.35%	1	
Absolutely sure	2.56%	1	4.35%	1	
This has already happened	0.00%	0	0.00%	0	
	**Parent 3**		**Staff 3**		**P Values**
**You will have to go to the hospital if you get the infection (n1 = 121; n2 = 32)**					**0.11**
**Answer**	**%**	**Count**	**%**	**Count**	
No chance	11.57%	14	9.38%	3	
Very small chance	70.25%	85	56.25%	18	
Medium chance	11.57%	14	21.88%	7	
High chance	2.48%	3	6.25%	2	
Very high chance	3.31%	4	0.00%	0	
Absolutely sure	0.83%	1	6.25%	2	
This has already happened	0.00%	0	0.00%	0	
	**Parent 1**		**Staff 1**		**P Values**
**Your child will have to go into quarantine independent of them being infected or not (n1 = 603; n2 = 379)**					**0.37**
**Answer**	**%**	**Count**	**%**	**Count**	
No chance	1%	6	0.53%	2	
Very small chance	16.58%	100	15.83%	60	
Medium chance	35.49%	214	40.63%	154	
High chance	24.05%	145	21.11%	80	
Very high chance	11.11%	67	12.14%	46	
Absolutely sure	2.82%	17	3.69%	14	
This has already happened	8.96%	54	6.07%	23	
	**Parent 2**		**Staff 2**		**P Values**
**Your child will have to go into quarantine independent of them being infected or not (n1 = 39; n2 = 23)**					**0.35**
**Answer**	**%**	**Count**	**%**	**Count**	
No chance	2.56%	1	0	0	
Very small chance	15.38%	6	30.43%	7	
Medium chance	46.15%	18	34.78%	8	
High chance	17.95%	7	26.09%	6	
Very high chance	0.00%	0	4.35%	1	
Absolutely sure	7.69%	3	0.00%	0	
This has already happened	10.26%	4	4.35%	1	
	**Parent 3**		**Staff 3**		**P Values**
**You will have to go into quarantine independent of you being infected or not (n1 = 120; n2 = 32)**					**0.083**
**Answer**	**%**	**Count**	**%**	**Count**	
No chance	4.17%	5	0.00%	0	
Very small chance	28.33%	34	43.75%	14	
Medium chance	31.67%	38	25%	8	
High chance	13.33%	16	18.75%	6	
Very high chance	0.83%	1	0.00%	0	
Absolutely sure	3.33%	4	9.38%	3	
This has already happened	18.33%	22	3.13%	1	
	**Parent 1**		**Staff 1**		**P Values**
**Your child will get infected and they will infect someone else (n1 = 607; n2 = 380)**					**0.98**
**Answer**	**%**	**Count**	**%**	**Count**	
No chance	0.99%	6	1.32%	5	
Very small chance	42.17%	256	41.58%	158	
Medium chance	46.46%	282	45.53%	173	
High chance	6.75%	41	7.11%	27	
Very high chance	2.64%	16	3.16%	12	
Absolutely sure	0.49%	3	0.53%	2	
This has already happened	0.49%	3	0.79%	3	
	**Parent 2**		**Staff 2**		**P Values**
**Your child will get infected and they will infect someone else (n1 = 39; n2 = 23)**					**0.67**
**Answer**	**%**	**Count**	**%**	**Count**	
No chance	2.56%	1	4.35%	1	
Very small chance	48.72%	19	43.48%	10	
Medium chance	38.46%	15	43.48%	10	
High chance	10.26%	4	4.35%	1	
Very high chance	0.00%	0	4.35%	1	
Absolutely sure	0.00%	0	0.00%	0	
This has already happened	0.00%	0	0.00%	0	
	**Parent 3**		**Staff 3**		**P Values**
**You will get infected and you will infect someone else (n1 = 121; n2 = 32)**					**0.33**
**Answer**	**%**	**Count**	**%**	**Count**	
No chance	8.26%	10	3.13%	1	
Very small chance	56.2%	68	62.5%	20	
Medium chance	26.45%	32	18.75%	6	
High chance	3.31%	4	12.5%	4	
Very high chance	0.00%	0	0.00%	0	
Absolutely sure	0.83%	1	0.00%	0	
This has already happened	4.96%	6	3.13%	1	

**Table 3.** Kindergarten to Grade 12 Parent and Staff Individual COVID-19 Disease Preventative Behavior

	**Parent 1**		**Parent 2**		**P Values**
**Wearing a face mask (n1 = 604; 39)**					**0.081**
**Answer**	**%**	**Count**	**%**	**Count**	
Not effective at all	1.16%	7	7.69%	3	
Hardly effective	3.15%	19	2.56%	1	
Somewhat effective	9.44%	57	10.26%	4	
Effective	28.31%	171	30.77%	12	
Very effective	57.95%	350	48.72%	19	

	**Staff 1**		**Staff 2**		**Staff 3**		**P Values**
**Wearing a face mask (n1 = 378; n2 = 23; n3 = 32)**							**0.29**
**Answer**	**%**	**Count**	**%**	**Count**	**%**	**Count**	
Not effective at all	0.00%	0	0.00%	0	0.00%	0	
Hardly effective	1.32%	5	4.35%	1	3.13%	1	
Somewhat effective	8.99%	34	0.00%	0	6.25%	2	
Effective	28.84%	109	39.13%	9	37.50%	12	
Very effective	60.85%	230	56.52%	13	53.13%	17	

	**Parent 1**		**Parent 2**		**P Values**
**Praying/performing religious ceremonies (n1 = 599; n2 = 39)**					**0.77**
**Answer**	**%**	**Count**	**%**	**Count**	
Not effective at all	70.12%	420	69.23%	27	
Hardly effective	13.19%	79	17.95%	7	
Somewhat effective	8.35%	50	10.26%	4	
Effective	5.51%	33	2.56%	1	
Very effective	2.84%	17	0.00%	0	

	**Staff 1**		**Staff 2**		**Staff 3**		**P Values**
**Praying/performing religious ceremonies (n1 = 375; n2 = 23; n3 = 32)**							**0.37**
**Answer**	**%**	**Count**	**%**	**Count**	**%**	**Count**	
Not effective at all	73.07%	274	69.57%	16	65.63%	21	
Hardly effective	14.67%	55	8.70%	2	15.63%	5	
Somewhat effective	7.20%	27	17.39%	4	9.38%	3	
Effective	2.93%	11	4.35%	1	9.38%	3	
Very effective	2.13%	8	0.00%	0	0.00%	0	

	**Parent 1**		**Parent 2**		**P Values**
**Washing their hands with soap or using hand sanitizer frequently (n1 = 602; n2 = 39)**					**0.13**
**Answer**	**%**	**Count**	**%**	**Count**	
Not effective at all	0.17%	1	2.56%	1	
Hardly effective	1.16%	7	2.56%	1	
Somewhat effective	9.97%	60	12.82%	5	
Effective	27.57%	166	20.51%	8	
Very effective	61.13%	368	61.54%	24	

	**Staff 1**		**Staff 2**		**Staff 3**		**P Values**
**Washing their hands with soap or using hand sanitizer frequently (n1 = 378; n2 = 23; n3 = 32)**							**0.41**
**Answer**	**%**	**Count**	**%**	**Count**	**%**	**Count**	
Not effective at all	0.53%	2	0.00%	0	0.00%	0	
Hardly effective	0.79%	3	0.00%	0	3.13%	1	
Somewhat effective	9.52%	36	8.70%	2	15.63%	5	
Effective	30.69%	116	47.83%	11	31.25%	10	
Very effective	58.47%	221	43.48%	10	50.00%	16	

	**Parent 1**		**Parent 2**		**P Values**
**Seeing a health care provider if they feel sick (n1 = 600; n2 = 39)**					**0.47**
**Answer**	**%**	**Count**	**%**	**Count**	
Not effective at all	4.5%	27	0.00%	0	
Hardly effective	7.17%	43	12.82%	5	
Somewhat effective	20.83%	125	20.51%	8	
Effective	33.33%	200	28.21%	11	
Very effective	34.17%	205	38.46%	15	

	**Staff 1**		**Staff 2**		**Staff 3**		**P Values**
**Seeing a health care provider if you feel sick (n1 = 378; n2 = 22; n3 = 31)**							**0.96**
**Answer**	**%**	**Count**	**%**	**Count**	**%**	**Count**	
Not effective at all	0.53%	2	0.00%	0	0.00%	0	
Hardly effective	0.79%	3	0.00%	0	3.13%	1	
Somewhat effective	9.52%	36	8.70%	2	15.63%	5	
Effective	30.69%	116	47.83%	11	31.25%	10	
Very effective	58.47%	221	43.48%	10	50.00%	16	

	**Parent 1**		**Parent 2**		**P Values**
**Seeing a health care provider if they feel healthy but you worry that they were exposed (n1 = 601; n2 = 39)**					**0.58**
**Answer**	**%**	**Count**	**%**	**Count**	
Not effective at all	16.31%	98	15.38%	6	
Hardly effective	22.13%	133	23.08%	9	
Somewhat effective	23.46%	141	30.77%	12	
Effective	21.3%	128	23.08%	9	
Very effective	16.81%	101	7.69%	3	

	**Staff 1**		**Staff 2**		**Staff 3**		**P Values**
**Seeing a health care provider if they feel healthy but you worry that they were exposed (n1 = 378; n2 = 23; n3 = 32)**							**0.68**
**Answer**	**%**	**Count**	**%**	**Count**	**%**	**Count**	
Not effective at all	11.64%	44	0.00%	0	15.63%	5	a
Hardly effective	18.52%	70	26.09%	6	12.50%	4	
Somewhat effective	29.10%	110	30.43%	7	31.25%	10	
Effective	25.40%	96	26.09%	6	31.25%	10	
Very effective	15.34%	58	17.39%	4	9.38%	3	

	**Parent 1**		**Parent 2**		**P Values**
**Avoiding public spaces, gatherings, and crowds (n1 = 603; n2 = 39)**					**0.091**
**Answer**	**%**	**Count**	**%**	**Count**	
Not effective at all	1.99%	12	2.56%	1	
Hardly effective	3.32%	20	10.26%	4	
Somewhat effective	11.61%	70	17.95%	7	
Effective	22.55%	136	23.08%	9	
Very effective	60.53%	365	46.15%	18	

	**Staff 1**		**Staff 2**		**Staff 3**		**P Values**
**Avoiding public spaces, gatherings, and crowds (n1 = 378; n2 = 23; n3 = 32)**							**0.0057**
**Answer**	**%**	**Count**	**%**	**Count**	**%**	**Count**	
Not effective at all	0.26%	1	0.00%	0	0.00%	0	
Hardly effective	0.79%	3	0.00%	0	0.00%	0	
Somewhat effective	5.82%	22	4.35%	1	12.50%	4	
Effective	17.46%	66	34.78%	8	43.75%	14	
Very effective	75.66%	286	60.87%	14	43.75%	14	

	**Parent 1**		**Parent 2**		**P Values**
**Avoiding contact with people who could be high-risk (n1 = 601; n2 = 38)**					**0.18**
**Answer**	**%**	**Count**	**%**	**Count**	
Not effective at all	1%	6	0.00%	0	
Hardly effective	2.16%	13	5.26%	2	
Somewhat effective	7.15%	43	13.16%	5	
Effective	23.13%	139	23.68%	9	
Very effective	66.56%	400	57.89%	22	

	**Staff 1**		**Staff 2**		**Staff 3**		**P Values**
**Avoiding contact with people who could be high-risk (n1 = 378; n2 = 23; n3 = 32)**							**0.62**
**Answer**	**%**	**Count**	**%**	**Count**	**%**	**Count**	
Not effective at all	0.26%	1	0.00%	0	0.00%	0	
Hardly effective	1.59%	6	0.00%	0	0.00%	0	
Somewhat effective	5.03%	19	13.04%	3	9.38%	3	
Effective	22.49%	85	17.39%	4	25.00%	8	
Very effective	70.63%	267	69.57%	16	65.63%	21	

	**Parent 1**		**Parent 2**		**P Values**
**Avoiding hospitals and clinics (n1 = 599; n2 = 39)**					**0.047**
**Answer**	**%**	**Count**	**%**	**Count**	
Not effective at all	5.18%	31	7.69%	3	
Hardly effective	14.19%	85	7.69%	3	
Somewhat effective	32.05%	192	33.33%	13	
Effective	23.71%	142	41.03%	16	
Very effective	24.87%	149	10.26%	4	

	**Staff 1**		**Staff 2**		**Staff 3**		**P Values**
**Avoiding hospitals and clinics (n1 = 377; n2 = 23; n3 = 32)**							**0.49**
**Answer**	**%**	**Count**	**%**	**Count**	**%**	**Count**	
Not effective at all	2.39%	9	4.35%	1	3.13%	1	
Hardly effective	12.47%	47	21.74%	5	21.88%	7	
Somewhat effective	27.06%	102	39.13%	9	28.13%	9	
Effective	29.71%	112	21.74%	5	25.00%	8	
Very effective	28.38%	107	13.04%	3	21.88%	7	

	**Parent 1**		**Parent 2**		**P Values**
**Avoiding restaurants, gyms, and/or salons (n1 = 603; n2 = 39)**					**0.44**
**Answer**	**%**	**Count**	**%**	**Count**	
Not effective at all	3.65%	22	5.13%	2	
Hardly effective	7.46%	45	12.82%	5	
Somewhat effective	23.55%	142	23.08%	9	
Effective	29.02%	175	33.33%	13	
Very effective	36.32%	219	25.64%	10	

	**Staff 1**		**Staff 2**		**Staff 3**		**P Values**
**Avoiding restaurants, gyms, and/or salons (n1 = 378; n2 = 23; n3 = 32)**							**0.26**
**Answer**	**%**	**Count**	**%**	**Count**	**%**	**Count**	
Not effective at all	0.79%	3	0.00%	0	0.00%	0	
Hardly effective	3.97%	15	4.35%	1	6.25%	2	
Somewhat effective	14.29%	54	17.39%	4	28.13%	9	
Effective	32.28%	122	21.74%	5	37.50%	12	
Very effective	48.68%	184	56.52%	13	28.13%	9	

	**Parent 1**		**Parent 2**		**P Values**
**Avoiding public transport (n1 = 601 ; n2 = 39)**					**0.083**
**Answer**	**%**	**Count**	**%**	**Count**	
Not effective at all	2.5%	15	7.69%	3	
Hardly effective	5.32%	32	12.82%	5	
Somewhat effective	23.13%	139	15.38%	6	
Effective	27.62%	166	25.64%	10	
Very effective	41.43%	249	38.46%	15	

	**Staff 1**		**Staff 2**		**Staff 3**		**P Values**
**Avoiding public transport (n1 = 378; n2 = 23; n3 = 32)**							**0.0044**
**Answer**	**%**	**Count**	**%**	**Count**	**%**	**Count**	
Not effective at all	0.00%	0	4.35%	1	0.00%	0	
Hardly effective	2.65%	10	0.00%	0	3.13%	1	
Somewhat effective	12.70%	48	26.09%	6	28.13%	9	
Effective	32.28%	122	39.13%	9	40.63%	13	
Very effective	52.38%	198	30.43%	7	28.13%	9	

	**Parent 1**		**Staff 1**		**P Values**
**Wearing a face mask (n1 = 604; n2 = 378)**					**0.086**
**Answer**	**%**	**Count**	**%**	**Count**	
Not effective at all	2.5%	15	7.69%	3	
Hardly effective	5.32%	32	12.82%	5	
Somewhat effective	23.13%	139	15.38%	6	
Effective	27.62%	166	25.64%	10	
Very effective	41.43%	249	38.46%	15	
	**Parent 2**		**Staff 2**		**P Values**
**Wearing a face mask (n1 = 39; n2 = 23)**					**0.36**
**Answer**	**%**	**Count**	**%**	**Count**	
Not effective at all	7.69%	3	0.00%	0	
Hardly effective	2.56%	1	4.35%	1	
Somewhat effective	10.26%	4	0.00%	0	
Effective	30.77%	12	39.13%	9	
Very effective	48.72%	19	56.52%	13	
	**Parent 1**		**Staff 1**		**P Values**
**Praying/performing religious ceremonies (n1 = 599; n2 = 375)**					**0.31**
**Answer**	**%**	**Count**	**%**	**Count**	
Not effective at all	70.12%	420	73.07%	274	
Hardly effective	13.19%	79	14.67%	55	
Somewhat effective	8.35%	50	7.20%	27	
Effective	5.51%	33	2.93%	11	
Very effective	2.84%	17	2.13%	8	
	**Parent 2**		**Staff 2**		**P Values**
**Praying/performing religious ceremonies (n1 = 39; n2 = 23)**					**0.64**
**Answer**	**%**	**Count**	**%**	**Count**	
Not effective at all	69.23%	27	69.57%	16	
Hardly effective	17.95%	7	8.70%	2	
Somewhat effective	10.26%	4	17.39%	4	
Effective	2.56%	1	4.35%	1	
Very effective	0	0	0.00%	0	
	**Parent 1**		**Staff 1**		**P Values**
**Washing their hands with soap or using hand sanitizer frequently (n1 = 602; n2 = 378)**					**0.67**
**Answer**	**%**	**Count**	**%**	**Count**	
Not effective at all	0.17%	1	0.53%	2	
Hardly effective	1.16%	7	0.79%	3	
Somewhat effective	9.97%	60	9.52%	36	
Effective	27.57%	166	30.69%	116	
Very effective	61.13%	368	58.47%	221	
	**Parent 2**		**Staff 2**		**P Values**
**Washing their hands with soap or using hand sanitizer frequently (n1 = 39; n2 = 23)**					**0.19**
**Answer**	**%**	**Count**	**%**	**Count**	
Not effective at all	2.56%	1	0.00%	0	
Hardly effective	2.56%	1	0.00%	0	
Somewhat effective	12.82%	5	8.70%	2	
Effective	20.51%	8	47.83%	11	
Very effective	61.54%	24	43.48%	10	
	**Parent 1**		**Staff 1**		**P Values**
**Seeing a health care provider if they feel sick (n1 = 600; n2 = 378)**					**0.097**
**Answer**	**%**	**Count**	**%**	**Count**	
Not effective at all	4.5%	27	1.59%	6	
Hardly effective	.7.17%	43	5.56%	21	
Somewhat effective	20.83%	125	22.49%	85	
Effective	33.33%	200	36.24%	137	
Very effective	34.17%	205	34.13%	129	
	**Parent 2**		**Staff 2**		**P Values**
**Seeing a health care provider if they feel sick (n1 = 39; n2 = 22)**					**0.33**
**Answer**	**%**	**Count**	**%**	**Count**	
Not effective at all	0	0	0.00%	0	
Hardly effective	12.82%	5	0.00%	0	
Somewhat effective	20.51%	8	22.73%	5	
Effective	28.21%	11	40.91%	9	
Very effective	38.46%	15	36.36%	8	
	**Parent 1**		**Staff 1**		**P Values**
**Seeing a health care provider if they feel healthy but you worry that they were exposed (n1 = 601; n2 = 378)**					**0.043**
**Answer**	**%**	**Count**	**%**	**Count**	
Not effective at all	16.31%	98	11.64%	44	
Hardly effective	.22.13%	133	18.52%	70	
Somewhat effective	23.46%	141	29.10%	110	
Effective	21.3%	128	25.40%	96	
Very effective	16.81%	101	15.34%	58	
	**Parent 2**		**Staff 2**		**P Values**
**Seeing a health care provider if they feel healthy but you worry that they were exposed (n1 = 39; n2 = 23)**					**0.30**
**Answer**	**%**	**Count**	**%**	**Count**	
Not effective at all	15.38%	6	0.00%	0	
Hardly effective	23.08%	9	26.09%	6	
Somewhat effective	30.77%	12	30.43%	7	
Effective	23.08%	9	26.09%	6	
Very effective	7.69%	3	17.39%	4	
	**Parent 1**		**Staff 1**		**P Values**
**Avoiding public spaces, gatherings, and crowds (n1 = 603; n2 = 378)**					**1.50E-06**
**Answer**	**%**	**Count**	**%**	**Count**	
Not effective at all	1.99%	12	0.26%	1	
Hardly effective	3.32%	20	0.79%	3	
Somewhat effective	11.61%	70	5.82%	22	
Effective	22.55%	136	17.46%	66	
Very effective	60.53%	365	75.66%	286	
	**Parent 2**		**Staff 2**		**P Values**
**Avoiding public spaces, gatherings, and crowds (n1 = 39; n2 = 23)**					**0.18**
**Answer**	**%**	**Count**	**%**	**Count**	
Not effective at all	2.56%	1	0.00%	0	
Hardly effective	10.26%	4	0.00%	0	
Somewhat effective	17.95%	7	4.35%	1	
Effective	23.08%	9	34.78%	8	
Very effective	46.15%	18	60.87%	14	
	**Parent 1**		**Staff 1**		**P Values**
**Avoiding contact with people who could be high-risk (n1 = 601; n2 = 378)**					**0.39**
**Answer**	**%**	**Count**	**%**	**Count**	
Not effective at all	1%	6	0.26%	1	
Hardly effective	2.16%	13	1.59%	6	
Somewhat effective	7.15%	43	5.03%	19	
Effective	23.13%	139	22.49%	85	
Very effective	66.56%	400	70.63%	267	
	**Parent 2**		**Staff 2**		**P Values**
**Avoiding contact with people who could be high-risk (n1 = 38; n2 = 23)**					**0.74**
**Answer**	**%**	**Count**	**%**	**Count**	
Not effective at all	0	0	0.00%	0	
Hardly effective	5.26%	2	0.00%	0	
Somewhat effective	13.16%	5	13.04%	3	
Effective	23.68%	9	17.39%	4	
Very effective	57.89%	22	69.57%	16	
	**Parent 1**		**Staff 1**		**P Values**
**Avoiding hospitals and clinics (n1 = 599; n2 = 377)**					**0.024**
**Answer**	**%**	**Count**	**%**	**Count**	
Not effective at all	5.18%	31	2.39%	9	
Hardly effective	14.19%	85	12.47%	47	
Somewhat effective	32.05%	192	27.06%	102	
Effective	23.71%	142	29.71%	112	
Very effective	24.87%	149	28.38%	107	
	**Parent 2**		**Staff 2**		**P Values**
**Avoiding hospitals and clinics (n1 = 39; n2 = 23)**					**0.37**
**Answer**	**%**	**Count**	**%**	**Count**	
Not effective at all	7.69%	3	4.35%	1	
Hardly effective	7.69	3	21.74%	5	
Somewhat effective	33.33%	13	39.13%	9	
Effective	41.03%	16	21.74%	5	
Very effective	10.26%	4	13.04%	3	
	**Parent 1**		**Staff 1**		**P Values**
**Avoiding restaurants, gyms, and/or salons (n1 = 603; n2 = 378)**					**2.42E-06**
**Answer**	**%**	**Count**	**%**	**Count**	
Not effective at all	3.65%	22	0.79%	3	
Hardly effective	7.46%	45	3.97%	15	
Somewhat effective	23.55%	142	14.29%	54	
Effective	29.02%	175	32.28%	122	
Very effective	36.32%	219	48.68%	184	
	**Parent 2**		**Staff 2**		**P Values**
**Avoiding restaurants, gyms, and/or salons (n1 = 39; n2 = 23)**					**0.18**
**Answer**	**%**	**Count**	**%**	**Count**	
Not effective at all	5.13%	2	0.00%	0	
Hardly effective	12.82%	5	4.35%	1	
Somewhat effective	23.08%	9	17.39%	4	
Effective	33.33%	13	21.74%	5	
Very effective	25.64%	10	56.52%	13	
	**Parent 1**		**Staff 1**		**P Values**
**Avoiding public transport (n1 = 601; n2 = 378)**					**5.95E-08**
**Answer**	**%**	**Count**	**%**	**Count**	
Not effective at all	2.5%	15	0.00%	0	
Hardly effective	5.32%	32	2.65%	10	
Somewhat effective	23.13%	139	12.70%	48	
Effective	27.62%	166	32.28%	122	
Very effective	41.43%	249	52.38%	198	
	**Parent 2**		**Staff 2**		**P Values**
**Avoiding public transport (n1 = 39; n2 = 23)**					**0.27**
**Answer**	**%**	**Count**	**%**	**Count**	
Not effective at all	7.69%	3	4.35%	1	
Hardly effective	12.82%	5	0.00%	0	
Somewhat effective	15.38%	6	26.09%	6	
Effective	25.64%	10	39.13%	9	
Very effective	38.46%	15	30.43%	7	

**Table 4.** Kindergarten to Grade 12 Parent and Staff Perception on COVID-19 Societal Policy

	**Parent 1**		**Parent 2**		**P Values**
**Close schools and daycares (n1 = 600; n2 = 39)**					**0.33**
**Answer**	**%**	**Count**	**%**	**Count**	
Not effective at all	13.67%	82	25.64%	10	
Hardly effective	22%	132	23.08%	9	
Somewhat effective	36.67%	220	33.33%	13	
Effective	17.83%	107	12.82%	5	
Very effective	9.83%	59	5.13%	2	
	**Staff 1**		**Staff 2**		**P Values**
**Close schools and daycares (n1 = 376; n2 = 23)**					**0.020**
**Answer**	**%**	**Count**	**%**	**Count**	
Not effective at all	3.72%	14	17.39%	4	
Hardly effective	14.63%	55	26.09%	6	
Somewhat effective	40.43%	152	21.74%	5	
Effective	28.19%	106	21.74%	5	
Very effective	13.03%	49	13.04%	3	
	**Parent 1**		**Parent 2**		**P Values**
**Close gyms (n1 = 599; n2 = 39)**					**0.024**
**Answer**	**%**	**Count**	**%**	**Count**	
Not effective at all	6.68%	40	15.38%	6	
Hardly effective	10.02%	60	7.69%	3	
Somewhat effective	22.7%	136	38.46%	15	
Effective	30.05%	180	23.08%	9	
Very effective	30.55%	183	15.38%	6	
	**Staff 1**		**Staff 2**		**P Values**
**Close gyms (n1 = 376; n2 = 23)**					**0.49**
**Answer**	**%**	**Count**	**%**	**Count**	
Not effective at all	1.33%	5	4.35%	1	
Hardly effective	5.32%	20	8.7%	2	
Somewhat effective	18.35%	69	13.04%	3	
Effective	34.84%	131	34.78%	8	
Very effective	40.16%	151	39.13%	9	
	**Parent 1**		**Parent 2**		**P Values**
**Close restaurants (n1 = 598; n2 = 39)**					**0.38**
**Answer**	**%**	**Count**	**%**	**Count**	
Not effective at all	7.53%	45	12.82%	5	
Hardly effective	14.55%	87	15.38%	6	
Somewhat effective	30.27%	181	38.46%	15	
Effective	25.25%	151	20.51%	8	
Very effective	22.41	134	12.82%	5	
	**Staff 1**		**Staff 2**		**P Values**
**Close restaurants (n1 = 376; n2 = 23)**					**0.95**
**Answer**	**%**	**Count**	**%**	**Count**	
Not effective at all	1.6%	6	0.00%	0	
Hardly effective	7.45%	28	4.35%	1	
Somewhat effective	26.33%	99	26.09%	6	
Effective	31.91%	120	39.13%	9	
Very effective	32.71%	123	30.43%	7	
	**Parent 1**		**Parent 2**		**P Values**
**Close all shops except for supermarkets and pharmacies (n1 = 599; n2 = 39)**					**0.40**
**Answer**	**%**	**Count**	**%**	**Count**	
Not effective at all	12.69%	76	20.51%	8	
Hardly effective	24.71%	148	20.51%	8	
Somewhat effective	28.21%	169	33.33%	13	
Effective	21.54%	129	20.51%	8	
Very effective	12.85%	77	5.13%	2	
	**Staff 1**		**Staff 2**		**P Values**
**Close all shops except for supermarkets and pharmacies (n1 = 376; n2 = 23)**					**0.37**
**Answer**	**%**	**Count**	**%**	**Count**	
Not effective at all	3.99%	15	4.35%	1	
Hardly effective	14.89%	56	17.39%	4	
Somewhat effective	31.38%	118	47.83%	11	
Effective	28.99%	109	21.74%	5	
Very effective	20.74%	78	8.7%	2	
	**Parent 1**		**Parent 2**		**P Values**
**Don’t allow visitors in hospitals, nursing homes and elderly homes (n1 = 599; n2 = 39)**					**0.26**
**Answer**	**%**	**Count**	**%**	**Count**	
Not effective at all	2.5%	15	0.00%	0	
Hardly effective	5.01%	30	12.82%	5	
Somewhat effective	26.71%	160	28.21%	11	
Effective	34.06%	204	35.9%	14	
Very effective	31.72%	190	23.08%	9	
	**Staff 1**		**Staff 2**		**P Values**
**Don’t allow visitors in hospitals, nursing homes and elderly homes (n1 = 375; n2 = 23)**					**0.0026**
**Answer**	**%**	**Count**	**%**	**Count**	
Not effective at all	0.53%	2	0.00%	0	
Hardly effective	3.73%	14	0.00%	0	
Somewhat effective	20.8%	78	60.87%	14	
Effective	43.2%	162	21.74%	5	
Very effective	31.73%	119	17.39%	4	
	**Parent 1**		**Parent 2**		**P Values**
**Oblige people aged 70+ or with a medical condition to stay at home except to do basic shopping or because urgent medical attention is required (n1 = 600; n2 = 39)**					**0.29**
**Answer**	**%**	**Count**	**%**	**Count**	
Not effective at all	0.83%	5	2.56%	1	
Hardly effective	6.33%	38	5.13%	2	
Somewhat effective	22%	132	33.33%	13	
Effective	34.83%	209	30.77%	12	
Very effective	36%	216	28.21%	11	
	**Staff 1**		**Staff 2**		**P Values**
**Oblige people aged 70+ or with a medical condition to stay at home except to do basic shopping or because urgent medical attention is required (n1 = 374; n2 = 23)**					**0.36**
**Answer**	**%**	**Count**	**%**	**Count**	
Not effective at all	1.07%	4	4.35%	1	
Hardly effective	4.28%	16	4.35%	1	
Somewhat effective	21.12%	79	30.43%	7	
Effective	42.25%	158	34.78%	8	
Very effective	31.28%	117	26.09%	6	
	**Parent 1**		**Parent 2**		**P Values**
**Oblige everyone who does not work in a crucial professional group (for example, people who work in healthcare, public transport, the food chain) stays at home except to do basic shopping or because urgent medical care is needed (n1 = 600, n2 = 38)**					**0.014**
**Answer**	**%**	**Count**	**%**	**Count**	
Not effective at all	9%	54	15.79%	6	
Hardly effective	15.33%	92	10.53%	4	
Somewhat effective	25.83%	155	28.95%	11	
Effective	28.83%	173	23.68%	9	
Very effective	21%	126	21.05%	8	
	**Staff 1**		**Staff 2**		**P Values**
**Oblige everyone who does not work in a crucial professional group (for example, people who work in healthcare, public transport, the food chain) stays at home except to do basic shopping or because urgent medical care is needed (n1 = 376, n2 = 23)**					**0.12**
**Answer**	**%**	**Count**	**%**	**Count**	
Not effective at all	2.93%	11	4.35%	1	
Hardly effective	6.91%	26	4.35%	1	
Somewhat effective	23.4%	88	47.83%	11	
Effective	36.97%	139	21.74%	5	
Very effective	29.79%	112	21.74%	5	
	**Parent 1**		**Parent 2**		**P Values**
**Mandatory mask wearing (n1 = 598; n2 = 38)**					**0.20**
**Answer**	**%**	**Count**	**%**	**Count**	
Not effective at all	3.18%	19	7.89%	3	
Hardly effective	4.18%	25	5.26%	2	
Somewhat effective	7.19%	43	13.16%	5	
Effective	1.89%	113	18.42%	7	
Very effective	66.56%	398	55.26%	21	
	**Staff 1**		**Staff 2**		**P Values**
**Mandatory mask wearing (n1 = 376; n2 = 23)**					**0.55**
**Answer**	**%**	**Count**	**%**	**Count**	
Not effective at all	0.27%	1	0.00%	0	
Hardly effective	2.39%	9	0.00%	0	
Somewhat effective	3.72%	14	4.35%	1	
Effective	16.49%	62	26.09%	6	
Very effective	77.13%	290	69.57%	16	
	**Parent 1**		**Staff 1**		**P Values**
**Close schools and daycares (n1 = 600; n2 = 376)**					**5.98E-09**
**Answer**	**%**	**Count**	**%**	**Count**	
Not effective at all	13.67%	82	3.72%	14	
Hardly effective	22%	132	14.63%	55	
Somewhat effective	36.67%	220	40.43%	152	
Effective	17.83%	107	28.19%	106	
Very effective	9.83%	59	13.03	49	
	**Parent 2**		**Staff 2**		**P Values**
**Close schools and daycares (n1 = 39; n2 = 23)**					**0.57**
**Answer**	**%**	**Count**	**%**	**Count**	
Not effective at all	25.64%	10	17.39%	4	
Hardly effective	23.08%	9	26.09%	6	
Somewhat effective	33.33%	13	21.74%	5	
Effective	12.82%	5	21.74%	5	
Very effective	5.13%	2	13.04%	3	
	**Parent 1**		**Staff 1**		**P Values**
**Close gyms (n1 = 599; n2 = 376)**					**3.88E-06**
**Answer**	**%**	**Count**	**%**	**Count**	
Not effective at all	6.68%	40	1.33%	5	
Hardly effective	10.02%	60	5.32%	20	
Somewhat effective	2.27%	136	18.35%	69	
Effective	30.05%	180	34.84%	131	
Very effective	30.55%	183	40.16%	151	
	**Parent 2**		**Staff 2**		**P Values**
**Close gyms (n1 = 39; n2 = 23)**					**0.059**
**Answer**	**%**	**Count**	**%**	**Count**	
Not effective at all	15.38%	6	4.35%	1	
Hardly effective	7.69%	3	8.7%	2	
Somewhat effective	38.46%	15	13.04%	3	
Effective	23.08%	9	34.78%	8	
Very effective	15.38%	6	39.13%	9	
	**Parent 1**		**Staff 1**		**P Values**
**Close restaurants (n1 = 598; n2 = 376)**					**5.28E-08**
**Answer**	**%**	**Count**	**%**	**Count**	
Not effective at all	7.53%	45	1.6%	6	
Hardly effective	14.55	87	7.45%	28	
Somewhat effective	30.27%	181	26.33%	99	
Effective	25.25%	151	31.91%	120	
Very effective	22.41%	134	32.71%	123	
	**Parent 2**		**Staff 2**		**P Values**
**Close restaurants (n1 = 39; n2 = 23)**					**0.060**
**Answer**	**%**	**Count**	**%**	**Count**	
Not effective at all	12.82%	5	0.00	0	
Hardly effective	15.38%	6	4.35%	1	
Somewhat effective	38.46%	15	26.09%	6	
Effective	20.51%	8	39.13%	9	
Very effective	12.82%	5	30.43%	7	
	**Parent 1**		**Staff 1**		**P Values**
**Close all shops except for supermarkets and pharmacies (n1 = 599; n2 = 376)**					**5.17E-09**
**Answer**	**%**	**Count**	**%**	**Count**	
Not effective at all	12.69%	76	3.99%	15	
Hardly effective	24.71%	148	14.89%	56	
Somewhat effective	28.21%	169	31.38%	118	
Effective	21.54%	129	28.99%	109	
Very effective	12.85%	77	20.74%	78	
	**Parent 2**		**Staff 2**		**P Values**
**Close all shops except for supermarkets and pharmacies (n1 = 39; n2 = 23)**					**0.43**
**Answer**	**%**	**Count**	**%**	**Count**	
Not effective at all	20.51%	8	4.35%	1	
Hardly effective	20.51%	8	17.39%	4	
Somewhat effective	33.33%	13	47.83%	11	
Effective	20.51%	8	21.74%	5	
Very effective	5.13%	2	8.7%	2	
	**Parent 1**		**Staff 1**		**P Values**
**Don’t allow visitors in hospitals, nursing homes and elderly homes (n1 = 599; n = 2 375)**					**0.0056**
**Answer**	**%**	**Count**	**%**	**Count**	
Not effective at all	2.5%	15	0.53%	2	
Hardly effective	5.01%	30	3.73%	14	
Somewhat effective	26.71%	160	20.8%	78	
Effective	34.06%	204	43.2%	162	
Very effective	31.72%	190	31.73%	119	
	**Parent 2**		**Staff 2**		**P Values**
**Don’t allow visitors in hospitals, nursing homes and elderly homes (n1 = 39; n2 = 23)**					**0.052**
**Answer**	**%**	**Count**	**%**	**Count**	
Not effective at all	0.00%	0	0.00%	0	
Hardly effective	12.82%	5	0.00%	0	
Somewhat effective	28.21%	11	60.87%	14	
Effective	35.9%	14	21.74%	5	
Very effective	23.08%	9	17.39%	4	
	**Parent 1**		**Staff 1**		**P Values**
**Oblige people aged 70+ or with a medical condition to stay at home except to do basic shopping or because urgent medical attention is required (n1 = 600; n2 = 374)**					**0.14**
**Answer**	**%**	**Count**	**%**	**Count**	
Not effective at all	0.83%	5	1.07%	4	
Hardly effective	6.33%	38	4.28%	16	
Somewhat effective	22%	132	21.12%	79	
Effective	34.83%	209	42.25%	158	
Very effective	36%	216	31.28%	117	
	**Parent 2**		**Staff 2**		**P Values**
**Oblige people aged 70+ or with a medical condition to stay at home except to do basic shopping or because urgent medical attention is required (n1 = 39; n2 = 23)**					**0.9902**
**Answer**	**%**	**Count**	**%**	**Count**	
Not effective at all	2.56%	1	4.35%	1	
Hardly effective	5.13%	2	4.35%	1	
Somewhat effective	33.33%	13	30.43%	7	
Effective	30.77%	12	34.78%	8	
Very effective	28.21%	11	26.09%	6	
	**Parent 1**		**Staff 1**		**P Values**
**Oblige everyone who does not work in a crucial professional group (for example, people who work in healthcare, public transport, the food chain) stays at home except to do basic shopping or because urgent medical care is needed (n1 = 600; n2 = 376)**					**6.94E-08**
**Answer**	**%**	**Count**	**%**	**Count**	
Not effective at all	9%	54	2.93%	11	
Hardly effective	15.33%	92	6.91%	26	
Somewhat effective	25.83%	155	23.4%	88	
Effective	28.83%	173	36.97%	139	
Very effective	21%	126	29.79%	112	
	**Parent 2**		**Staff 2**		**P Values**
**Oblige everyone who does not work in a crucial professional group (for example, people who work in healthcare, public transport, the food chain) stays at home except to do basic shopping or because urgent medical care is needed (n1 = 38; n2 = 23)**					**0.49**
**Answer**	**%**	**Count**	**%**	**Count**	
Not effective at all	15.79%	6	4.35%	1	
Hardly effective	10.53	4	4.35%	1	
Somewhat effective	28.95%	11	47.83%	11	
Effective	23.68%	9	21.74%	5	
Very effective	21.05%	8	21.74%	5	
	**Parent 1**		**Staff 1**		**P Values**
**Mandatory mask wearing (n1 = 596; n2 = 376)**					**0.00015**
**Answer**	**%**	**Count**	**%**	**Count**	
Not effective at all	3.18%	19	0.27%	1	
Hardly effective	4.18%	25	2.39%	9	
Somewhat effective	7.19%	43	3.72%	14	
Effective	18.9%	113	16.49%	62	
Very effective	66.56%	398	77.13%	290	
	**Parent 2**		**Staff 2**		**P Values**
**Mandatory mask wearing (n1 = 38; n2 = 23)**					**0.39**
**Answer**	**%**	**Count**	**%**	**Count**	
Not effective at all	7.89%	3	0.00%	0	
Hardly effective	5.26%	2	0.00%	0	
Somewhat effective	13.16%	5	4.35%	1	
Effective	18.42%	7	26.09%	6	
Very effective	55.26%	21	69.57%	16	

**Table 5.** K-12 Parent and Staff Adherence to COVID-19 Policy

	**Parent 3**		**Staff 3**		**P Values**
**I have practiced recommend hand washing practice for at least 20 seconds (n1 = 119; n2 = 31)**					**0.35**
**Answer**	**%**	**Count**	**%**	**Count**	
Never	0.84%	1	0.00%	0	
rarely	0.00%	0	3.23%	1	
Sometimes	13.45%	16	6.45%	2	
Often	45.38%	54	45.16%	14	
Always	40.34%	48	45.16%	14	
	**Parent 3**		**Staff 3**		**P Values**
**I have practiced avoiding touching eyes, nose, and mouth with unwashed hands (n1 = 120; n2 = 31)**					**0.31**
**Answer**	**%**	**Count**	**%**	**Count**	
Never	0.00%	0	0.00%	0	
rarely	2.5%	3	6.45%	2	
Sometimes	24.17%	29	12.9%	4	
Often	42.5%	51	51.61%	16	
Always	30.83%	37	29.03%	9	
	**Parent 3**		**Staff 3**		**P Values**
**I have practice use of disinfectants to clean hands when soap and water was not available for washing hands (n1 = 119; n2 = 31)**					**0.61**
**Answer**	**%**	**Count**	**%**	**Count**	
Never	1.68%	2	0.00%	0	
rarely	3.36%	4	0.00%	0	
Sometimes	9.24%	11	12.9%	4	
Often	33.61%	40	45.16%	14	
Always	52.1%	62	41.94%	13	
	**Parent 3**		**Staff 3**		**P Values**
**I have been staying home/isolating when I was sick or when I had a cold (n1 = 120; n2 = 30)**					**0.89**
**Answer**	**%**	**Count**	**%**	**Count**	
Never	4.17%	5	0.00%	0	
rarely	1.67%	2	0.00%	0	
Sometimes	10%	12	10%	3	
Often	22.5%	27	20%	6	
Always	61.67%	74	70%	21	
	**Parent 3**		**Staff 3**		**P Values**
**I have been practicing physical distancing at least 6-ft away from others (n1 = 120; n2 = 31)**					**0.46**
**Answer**	**%**	**Count**	**%**	**Count**	
Never	0.00%	0	0.00%	0	
rarely	4.17%	5	0.00%	0	
Sometimes	14.17%	17	9.68%	3	
Often	45.83%	55	61.29%	19	
Always	35.83%	43	29.03%	9	
	**Parent 3**		**Staff 3**		**P Values**
**I have been practicing covering my mouth and nose when I cough or sneeze (n1 = 120; n2 = 31)**					**0.67**
**Answer**	**%**	**Count**	**%**	**Count**	
Never	0.00%	0	0.00%	0	
rarely	0.00%	0.00%	0.00%	0	
Sometimes	1.67%	2	3.23%	1	
Often	10.83%	13	12.9%	4	
Always	8.75%	105	83.87%	26	
	**Parent 3**		**Staff 3**		**P Values**
**I have been practicing disinfecting surfaces that belong to me. (n1 = 120; n2 = 31)**					**0.20**
**Answer**	**%**	**Count**	**%**	**Count**	
Never	4.17%	5	0.00%	0	
rarely	10.83%	13	16.13%	5	
Sometimes	24.17%	29	9.68%	3	
Often	39.17%	47	38.71%	12	
Always	21.67%	26	35.48%	11	
	**Parent 3**		**Staff 3**		**P Values**
**I have been wearing a face mask when I go to crowding area (n1 = 120; n2 = 31)**					**0.64**
**Answer**	**%**	**Count**	**%**	**Count**	
Never	0.00%	0	0.00%	0	
rarely	4.17%	5	0.00%	0	
Sometimes	4.17%	5	0.00%	0	
Often	14.17%	17	16.13%	5	
Always	77.5%	93	83.87%	26	
	**Parent 3**		**Staff 3**		**P Values**
**I am using antibiotics for preventive purposes (n1 = 119; n2 = 30)**					**0.86**
**Answer**	**%**	**Count**	**%**	**Count**	
Never	85.71%	102	90%	27	
rarely	6.72%	8	6.67%	2	
Sometimes	2.52%	3	0.00%	0	
Often	1.68%	2	3.33%	1	
Always	3.36%	4	0.00%	0	
	**Parent 3**		**Staff 3**		**P Values**
**I am using Herbal supplements for preventive purposes (n1 = 119; n2 = 31)**					**0.67**
**Answer**	**%**	**Count**	**%**	**Count**	
Never	65.55%	78	80.65%	25	
rarely	12.61%	15	6.45%	2	
Sometimes	10.92%	13	9.68%	3	
Often	7.56%	9	3.23%	1	
Always	3.36%	4	0.00%	0	
	**Parent 3**		**Staff 3**		**P Values**
**I am using homeopathic remedies for preventive purposes (n1 = 119; n2 = 30)**					**0.25**
**Answer**	**%**	**Count**	**%**	**Count**	
Never	68.07%	81	83.33%	25	
rarely	14.29%	17	3.33%	1	
Sometimes	10.08%	12	10%	3	
Often	5.88%	7	0.00%	0	
Always	1.68%	2	3.33%	1	
	**Parent 3**		**Staff 3**		**P Values**
**I am disinfecting my mobile phone with alcohol-based sanitizer (n1 = 120; n2 = 31)**					**0.78**
**Answer**	**%**	**Count**	**%**	**Count**	
Never	2.75%	33	22.58%	7	
rarely	18.33%	22	16.13%	5	
Sometimes	26.67%	32	38.71%	12	
Often	18.33%	22	1.29%	4	
Always	9.17%	11	9.68%	3	
	**Parent 3**		**Staff 3**		**P Values**
**I am eating Garlic, Ginger, Lemon, Feto (n1 = 120; n2 = 31)**					**0.64**
**Answer**	**%**	**Count**	**%**	**Count**	
Never	33.33%	40	48.39%	15	
rarely	18.33%	22	16.13%	5	
Sometimes	28.33%	34	19.35%	6	
Often	16.67%	20	12.9%	4	
Always	3.33%	4	3.23%	1	
