# Supplementary material for: A Proposal of Utilizing Six Types of Involvement Model to Guide Kindergarten to 12th Grade School Parental Communication and Support During a Pandemic
Source: Med Res Arch. Author manuscript; Available in PMC 2024 Aug 8. (PMC11309007; doi:10.18103/mra.v12i4.5178)
Supplement: 1 [file NIHMS1994435-supplement-1.pdf]

Supplemental Information

Appendix I. Codebook

Code Name	Abbreviation	Definition
Contextual Factors		
Role & Responsibilities	Rol	Refers to the different roles of the interviewees and/or the individuals mentioned in the interviews.
Setting	Set	Refers to school information
Operation		
Resources	Res_Fin	Refers to the resources and financial support school received for pandemic relief effort
Communication	Comm	To describe how COVID-19-related communications, including messages being communicated, communication channels, etc.
Safety Protocol	Safety_Pro	To describe how COVID-19 impacted routine school protocols and activities, including prevention protocols, etc.
COVID-19 Epidemiology	Epi	To describe the COVID-19 prevalence and transmission situation in school or local community
Mental Health	Men_Heal	To describe school members' mental health status during the pandemic
Attitudes	Att	To describe community members' perceptions and attitudes regarding the pandemic and school operation during the pandemic
Behavior	Beh	To describe school members' behaviors and adherence to the protocols
Overarching Codes		
Successes/Facilitators	Suc_Fac	Things that worked well, positive support.
Challenges/Barriers	Chal_Bar	Things that didn't work well
Recommendations	Rec	Recommenation for the project or for research team.
Great Quote	Grt_Qot	Anything that might be particularly useful, either to the project or for a future paper/presentation.

Appendix II. Parents and Staff Demographic Information

Parents and Staff Demographics

Gender (Table IIA)

Among the 1,221 total participants in the survey, 782 were parents (64.0%), and 439 (36.0%) were staff members. There were 858 (70.3%) female participants, of whom 573 (46.9%) were parents and 285 (23.3%) were staff members. There are 352 male participants (28.8%), with 204 (16.7%) being parents and 148 (12.1%) identified as staff.

Female participants outnumbered males in both the parent and staff member categories in all three rounds. Among parents, female respondents were the largest group in Round 1 (454/620, 72.2%), and the same applied to staff members (244/383, 63.7%). No significant differences were observed among parents in all three rounds (P=0.29), and the same held true for staff members (P=0.17).

When comparing the numbers in each round, parents consistently outnumbered staff members. In Round 1, for instance, a total of 620 (50.8%) parents participated compared to only 383 (31.4%). Female participants who were parents (454/620, 73.2%) and staff members (244/383, 65.71%) exceeded male respondents.

Education (Table IIB)

Regarding educational attainment, most parents (559/867, 64.5%) and staff members (288/439, 65.6%) had pursued post-graduate studies. In Round 1, 62.3% of parents (387/621) attended graduate school, whereas the corresponding figures were 55.3% (86/123) in Round 2 and 69.9% (86/123) in Round 3. No significant differences were detected among parents for all educational attainments in all three rounds (P=0.43).

Among staff member respondents who participated in Round 1 of the survey, 66.3% (254/383) pursued post-graduate studies. This trend persisted in Round 2 (14/23, 60.9%) and Round 3 (20/22, 60.6%). No significant differences were observed among staff members in terms of educational attainments across all three rounds (P=0.28). Likewise, Round 2 and 3, no significant differences between parents and staff were found (Round 1 P=0.03; Round 2 P=0.16; Round 3 P=0.31).

Race (Table IIC)

In terms of race, most parents (617/817, 75.56%) and staff members (404/455, 88.8%) identified as White. This racial composition remained consistent across all three survey rounds for both categories. Additionally, other racial groups, such as Asian, Black/African American, and Hispanic/Latino/Latinx, were also represented. Statistical analysis did not reveal significant differences among parents in terms of racial composition in all three rounds (P=0.72). In Round 1, there are significantly more Asian participants amongst parents (66/646, 10.22%; P<0.005). No significant differences in racial composition were found between parents and staff members in Round 2 (P=0.14), or Round 3 (P=0.14).

Tables. Parent and Faculty Survey Data

Table IIA. Gender

	Parent 1		Parent 2		Parent 3		P Values
	(n1 = 620; n2 = 39; n3 = 123)						0.28
Survey	Count	%	Count	%	Count	%	
Female	454	73.23%	34	87.18%	85	69.11 %	
Male	162	26.13%	5	12.82%	37	30.08 %	
Non-binary/third gender	0	0.00%	0	0.00%	0	0.00%	
Prefer to self-describe	1	0.16%	0	0.00%	0	0.00%	
Prefer not to say	3	0.48%	0	0.00%	1	0.81%	
	Faculty 1		Faculty 2		Faculty 3		P Values
	(n1 = 383; n2 = 23; n3 = 33)						0.17
Survey	Count	%	Count	%	Count	%	
Female	454	73.23%	34	87.18%	85	69.11 %	
Male	162	26.13%	5	12.82%	37	30.08 %	
Non-binary/third gender	0	0.00%	0	0.00%	0	0.00%	
Prefer to self-describe	1	0.16%	0	0.00%	0	0.00%	
Prefer not to say	3	0.48%	0	0.00%	1	0.81%	

	Parent 1		Faculty 1		P Values
	(n1 = 620; n2 = 383)				0.0014
**Survey**	**Count**	**%**	**Count**	**%**	
Female	454	73.23%	244	63.71%	
Male	162	26.13%	134	34.99%	
Non-binary/third gender	0	0.00%	3	0.78%	
Prefer to self-describe	1	0.16%	0	0.00%	

Prefer not to say	3	0.48%	2	0.52%	
	Parent 2		Faculty 2		P Values
	(n1 = 39; n2 = 23)				0.032
**Survey**	**Count**	**%**	**Count**	**%**	
Female	34	87.18%	14	60.87%	
Male	5	12.82%	8	34.78%	
Non-binary/third gender	0	0.00%	0	0.00%	
Prefer to self-describe	0	0.00%	0	0.00%	
Prefer not to say	0	0.00%	1	4.35%	
	Parent 3		Faculty 3		P Values
	(n1 = 123; n2 = 33)				0.36
**Survey**	**Count**	**%**	**Count**	**%**	
Female	85	69.11%	27	81.82%	
Male	37	30.08%	6	18.18%	
Non-binary/third gender	0	0.00%	0	0.00%	
Prefer to self-describe	0	0.00%	0	0.00%	
Prefer not to say	1	0.81%	0	0.00%	

Table IIB. Education

	Parent 1		Parent 2		Parent 3		P Values
	(n1 = 621; n2 = 123; n3 = 123)						0.059
**Survey**	**Count**	**%**	**Count**	**%**	**Count**	**%**	
Grades 1-8	0	0.00%	0	0.00%	0	0.00%	
Grades 9-11/Some high school	1	0.16%	2	2.63%	2	1.63%	
Grade 12/Completed high school or GED	6	0.97%	1	2.63%	1	0.81%	
Some college, Associates, Technical Degree	29	4.67%	6	2.63%	6	4.88%	
Bachelor's Degree	198	31.88%	28	36.84%	28	22.76%	
Any post-graduate studies	387	62.32%	86	55.26%	86	69.92%	

	Faculty 1		Faculty 2		Faculty 3		P Values
	(n1 = 383; n2 = 23; n3 = 33)						0.26
**Survey**	**Count**	**%**	**Count**	**%**	**Count**	**%**	
Grades 1-8	0	0.00%	0	0.00%	0	0.00%	
Grades 9-11/Some high school	0	0.00%	0	0.00%	0	0.00%	
Grade 12/Completed high school or GED	7	1.83%	2	8.70%	0	0.00%	
Some college, Associates, Technical Degree	28	7.31%	2	8.70%	5	15.15%	
Bachelor's Degree	94	24.54%	5	21.74%	8	24.24%	
Any post-graduate studies	254	66.32%	14	60.87%	20	60.61%	

	Parent 1		Faculty 1		P Values
	(n1 = 621; n2 = 383)				0.029
**Survey**	**Count**	**%**	**Count**	**%**	
Grades 1-8	0	0.00%	0	0.00%	
Grades 9-11/Some high school	1	0.16%	0	0.00%	
Grade 12/Completed high school or GED	6	0.97%	7	1.83%	
Some college, Associates, Technical Degree	29	4.67%	28	7.31%	
Bachelor's Degree	198	31.88%	94	24.54%	
Any post-graduate studies	387	62.32%	254	66.32%	
	Parent 2		Faculty 2		P Values
	(n1 = 123; n2 = 23)				0.16
**Survey**	**Count**	**%**	**Count**	**%**	
Grades 1-8	0	0.00%	0	0.00%	
Grades 9-11/Some high school	2	2.63%	0	0.00%	
Grade 12/Completed high school or GED	1	2.63%	2	8.70%	

Some college, Associates, Technical Degree	6	2.63%	2	8.70%	
Bachelor's Degree	28	36.84%	5	21.74%	
Any post-graduate studies	86	55.26%	14	60.87%	
	Parent 3		Faculty 3		P Values
	(n1 = 123; n2 = 33)				0.31
**Survey**	**Count**	**%**	**Count**	**%**	
Grades 1-8	0	0.00%	0	0.00%	
Grades 9-11/Some high school	2	1.63%	0	0.00%	
Grade 12/Completed high school or GED	1	0.81%	0	0.00%	
Some college, Associates, Technical Degree	6	4.88%	5	15.15%	
Bachelor's Degree	28	22.76%	8	24.24%	
Any post-graduate studies	86	69.92%	20	60.61%	

Table IIC. Race

	Parent 1		Parent 2		Parent 3		P Values
	(n1 = 646; n2 = 43; n3 = 128)						0.72
**Survey**	**Count**	**%**	**Count**	**%**	**Count**	**%**	
American Indian/Alaska Native	1	0.15%	0	0.00%	1	0.78%	
Asian	66	10.22%	6	13.95%	18	14.06%	
Black/African American	23	3.56%	2	4.65%	6	4.69%	
Hispanic/Latino/Latinx	25	3.87%	2	4.65%	9	7.03%	
Native Hawaiian or Other Pacific Islander	2	0.31%	0	0.00%	0	0.00%	
White	498	77.09%	31	72.09%	88	68.75%	
Other	16	2.48%	1	2.33%	1	0.78%	
Prefer not to say	15	2.32%	1	2.33%	5	3.91%	
	Faculty 1		Faculty 2		Faculty 3		P Values
	(n1 = 399; n2 = 23; n3 = 33)						0.023

Survey	Count	%	Count	%	Count	%	
American Indian/Alaska Native	0	0.00%	0	0.00%	0	0.00%	
Asian	4	1.00%	1	4.35%	3	9.09%	
Black/African American	9	2.26%	0	0.00%	0	0.00%	
Hispanic/Latino/Latinx	14	3.51%	0	0.00%	1	3.03%	
Native Hawaiian or Other Pacific Islander	3	0.75%	1	4.35%	0	0.00%	
White	358	89.72%	19	82.61%	27	81.82%	
Other	6	1.50%	0	0.00%	1	3.03%	
Prefer not to say	5	1.25%	2	8.70%	1	3.03%	

	Parent 1		Faculty 1		P Values
	(n1 = 646; n2 = 399)				0.00000059
Survey	Count	%	Count	%	
American Indian/Alaska Native	1	0.15%	0	0.00%	
Asian	66	10.22%	4	1.00%	
Black/African American	23	3.56%	9	2.26%	
Hispanic/Latino/Latinx	25	3.87%	14	3.51%	
Native Hawaiian or Other Pacific Islander	2	0.31%	3	0.75%	
White	498	77.09%	358	89.72%	
Other	16	2.48%	6	1.50%	
Prefer not to say	15	2.32%	5	1.25%	
	Parent 2		Faculty 2		P Values
	(n1 = 43; n2 = 23)				0.34
Survey	Count	%	Count	%	
American Indian/Alaska Native	0	0.00%	0	0.00%	
Asian	6	13.95%	1	4.35%	
Black/African American	2	4.65%	0	0.00%	
Hispanic/Latino/Latinx	2	4.65%	0	0.00%	

Native Hawaiian or Other Pacific Islander	0	0.00%	1	4.35%	
White	31	72.09%	19	82.61%	
Other	1	2.33%	0	0.00%	
Prefer not to say	1	2.33%	2	8.70%	
	Parent 3		Faculty 3		P Values
	(n1 = 128; n2 = 33)				0.62
Survey	Count	%	Count	%	
American Indian/Alaska Native	1	0.78%	0	0.00%	
Asian	18	14.06%	3	9.09%	
Black/African American	6	4.69%	0	0.00%	
Hispanic/Latino/Latinx	9	7.03%	1	3.03%	
Native Hawaiian or Other Pacific Islander	0	0.00%	0	0.00%	
White	88	68.75%	27	81.82%	
Other	1	0.78%	1	3.03%	
Prefer not to say	5	3.91%	1	3.03%	
